# Occupational Therapy in Fatigue Management in Multiple Sclerosis: An Umbrella Review

**DOI:** 10.1155/2019/2027947

**Published:** 2019-03-21

**Authors:** Angela Salomè, Tullia Sasso D'Elia, Giorgia Franchini, Valter Santilli, Teresa Paolucci

**Affiliations:** Complex Unit of Physical Medicine and Rehabilitation, Policlinico Umberto I Hospital, Sapienza University of Rome, Italy

## Abstract

*Background. *Fatigue is one of the most invalidant symptoms of Multiple Sclerosis (MS) that negatively affects occupational and work performance and social participation. Occupational therapy (OT) assessment and treatment of impairments related to fatigue can have a significant and positive impact on the quality of life.* Methods.* An umbrella review has been carried out to provide rehabilitative decision makers in healthcare with insight into the role of OT in fatigue management in Multiple Sclerosis. The question is, what type of treatment provided by occupational therapist is more effective in reducing fatigue in Multiple Sclerosis? A search of literature published until June 2018 was undertaken by three independent reviewers using PubMed, PEDro, and Cochrane Library database including systematic reviews and meta-analyses of the last 10 years.* Results*. 10 studies were selected (5 systematic reviews, 1 meta-analysis, 3 reviews, and 1 guideline).* Conclusions.* Fatigue management programs have moderate evidence; other strategies such as OT strategies and telerehabilitation show low evidence.

## 1. Introduction

Multiple Sclerosis (MS) is characterized by significant mental and physical symptoms, specially muscle weakness, abnormal walking mechanics, balance problems, spasticity, depression, cognitive impairment, and fatigue. Fatigue is defined by the Multiple Sclerosis Council for Clinical Practice Guidelines as “*a subjective lack of physical or mental energy that is perceived by the individual or caregiver to interfere with usual and desired activities*” [1]. Almost 80% of patients with MS report fatigue in the first year of disease onset [2]. Fatigue is a highly prevalent symptom in the early stage of the disease, with 55% of patients describing it as one of the worst symptoms they experience [3]. With disease advancing 95% of patients report fatigue [4].

In spite of its high prevalence, the pathophysiology of MS fatigue is not well understood and multiple mechanisms seem implicated in this setting [5]. Patients with MS frequently decrease physical activity due to the fear from worsening the symptoms and above all because of the easy fatigue, and this can result in reconditioning [6].

Fatigue in MS impacts on quality of life (QoL), reduces the capacity to perform activities of daily living, and affects occupational and work performance and social participation [18–20].

According to the latest good rehabilitative practices in MS patients the rehabilitative interventions are fundamental to improve fatigue [21]. Occupational therapy (OT) assessment and treatment of impairments related to movement can have a significant and positive impact on the quality of life [14, 15, 22]. The primary purpose of occupational therapy is to enable individuals to participate in self-care, work, and leisure activities that they want or need to perform [23]. With the increase in the number of systematic reviews available with respect to the efficacy of occupational therapy in SM patients regarding fatigue management, we have carried out an umbrella review to provide rehabilitative decision makers in healthcare with insight into the role of OT.

Then, the aim of this umbrella review is to assess the efficacy of the occupational therapy in the management of fatigue in people with Multiple Sclerosis. In particular we want to respond to the following question:* what type of treatment provided by occupational therapist is more effective in reducing fatigue in Multiple Sclerosis?*

## 2. Methods

A search of literature published until June 2018 was undertaken by three independent reviewers using PubMed, PEDro, and Cochrane Library database. We used the following filters: “systematic reviews”, “reviews”, “meta-analysis”, and “practice guideline”. We used the following key words: “multiple sclerosis”, “fatigue”, “occupational therapy”, and “energy conservation”, with Boolean operators “AND” and “OR”.

A search for gray literature from government and nongovernment organizations was performed using Google Scholar. The bibliographies of identified articles were analysed for additional references. We included research syntheses conducted within the past 10 years.

### 2.1. Inclusion Criteria

We included all reviews, systematic reviews, and meta-analyses of the last 10 years that concerned occupational and vocational rehabilitation treatment of fatigue in Multiple Sclerosis patients (Table 1).

Population must have diagnosis of Multiple Sclerosis at every stage of severity of disease, being male and female and aged >18 years old. Interventions may be occupational rehabilitation treatment such as Fatigue Management Course and Energy Conservation compared with other pharmacological and nonpharmacological intervention and the outcome should be the reduction of fatigue in daily live activities by common fatigue scales.

### 2.2. Exclusion Criteria

We excluded all RCTs (randomized controlled trials) or experimental studies or reviews published before 10 years, articles in other languages than English, articles without full text available, and articles that did not mention occupational rehabilitation treatment or vocational therapy. We also excluded reviews that incorporate theoretical studies or published opinion as their primary source of evidence (Table 1).

### 2.3. Study Selection and Data Extraction

Two authors independently screened all abstracts and articles for inclusion and appropriateness based on selection criteria. Any disagreement regarding the possible inclusion/exclusion of a study was resolved by a final consensus. Data extraction was conducted independently, using a standard pro forma. Information obtained from all reviews included publication and search date, objectives, characteristics of included studies and study subjects, intervention, findings/patient outcomes in the review, and limitations [24].

Systematic reviews and meta-analyses that were eligible for inclusion in this umbrella review were assessed for methodological quality using the Assessment of Multiple Systematic Reviews (AMSTAR) appraisal tool [25, 26] (Table 2).

## 3. Results

**Figure 1:**
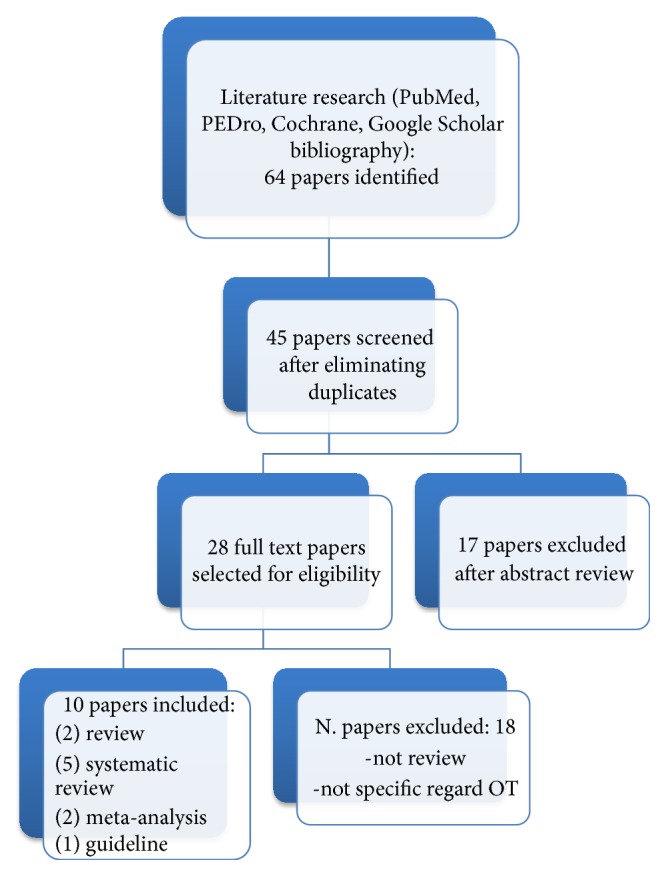
Flow chart that shows selection process.

The electronic database search retrieved 64 published articles on fatigue in MS; 45 articles met title inclusion criteria, of which 28 articles met the abstract inclusion criteria and went on to full-text review. 3 articles that met the abstract inclusion criteria were identified from the bibliographies of relevant articles. Overall, 10 studies were selected (5 systematic reviews, 1 meta-analysis, 3 reviews, and 1 guideline) which fulfilled the inclusion criteria for this review. The study selection process is summarized in the flow diagram shown in Figure 1. The characteristics of included reviews are summarized in Table 3.

NICE published a recent guideline about the management of Multiple Sclerosis in adults. This guideline was developed in 2014 by the National Clinical Guideline Centre, which is based in the Royal College of Physicians [7]. The Collaborating Centre worked with a Guideline Development Group, including healthcare professionals (consultants, occupational therapist, GPs, and nurses), patients and carers, and technical staff, who reviewed the evidence and drafted the recommendations. All searches were conducted in MEDLINE, Embase, and the Cochrane Library and were updated for the final time on February 2014. They evaluated the quality of evidence with GRADE. They focused mainly on the assessment of fatigue and on the necessity of a multidisciplinary team for the problem management. They dealt also with fatigue management programs. This guideline shows some limitations like the low quality of the original evidence.

Khan [8] conducted a recent systematic review of the rehabilitation of Multiple Sclerosis comprehensive of OT rehabilitation. The authors included all systematic reviews that assessed effectiveness of organized rehabilitation, both uni- and multidisciplinary. They searched literature until 2016 and finally included 15 Cochrane reviews and 24 other reviews. They used the validated and commonly used tools to assess the methodology (AMSTAR) and quality of evidence (GRADE) of these reviews. The topic concerns multidisciplinary rehabilitation, FACETS programs, and energy conservation.

The review is the only one that approaches also the vocational rehabilitation programs to help MS patients who were experiencing work instability to obtain and maintain employment. This review has a high level of quality, with a AMSTAR score of 10, but also some limitations like the lack of methodologically robust trials, and there is a probable bias because 4 reviews included in the analysis are of the same research group.

Tur [9] conducted a review published in 2016 about the fatigue in MS. The author did not explain clearly the study design and the selection criteria and the review obtained only a AMSTAR score of 2, but the author talked about different aspects of fatigue: clinical aspect, scale of measure, possible causes and common triggers, and finally the pharmacological and nonpharmacological management like energy conservation, the FACETS (Fatigue: Applying Cognitive behavioural and Energy effectiveness Techniques to life Style) program, and the EXIM (pragmatic EXercise Intervention for people with MS) program.

Hourihan [10] published in 2015 a review that explores assessment and measurement of fatigue, evidence-based pharmacological and nonpharmacological treatments for managing fatigue, and the role of MS specialist, nurses, and therapists. The methodology used to select literature and extract data is not clear (AMSTAR 2). The author discussed energy conservation, FACETS program, and EXIM program.

Khan et al. [11] have carried out a search of the literature published until June 2014 using MEDLINE, Embase, PubMed, and Cochrane Library database, including 27 studies of high level (12 RCTs, 12 SR, 2 CCT, and 1 other), 6 of which are about OT. They examined if a structured fatigue management program based on psychological approaches delivered by health professionals can be effective in reducing fatigue severity and increasing fatigue self-efficacy for people with MS. It is a high level review (AMSTAR 10), but the authors stated that the number of high level studies is low.

Asano et al. [12] performed a literature search in PubMed, Embase, Cinahl, and PsycINFO till 2013; they found 17 exercise intervention studies and 21 behaviour change intervention studies. They discussed major types of rehabilitation interventions (e.g., exercise or physical therapy, educational, self-management program, and psychotherapy) that are commonly used as traditional rehabilitation settings by rehabilitation professionals (e.g., occupational and physical therapists, nurses, psychologists, and physiatrists). The review published in 2015 is well structured, AMSTAR 10, but many studies analysed have small sample size.

Asano and Finlayson [13] published in 2014 a meta-analysis of mild-high quality (AMSTAR 6); the authors researched literature using PubMed, Embase, and Cinahl till August 2013. They selected 18 rehabilitation trials (ten exercise intervention trials and eight educational intervention trials) and 7 pharmacological trials targeting fatigue. They described different approaches: fatigue management program, energy conservation course, Cognitive Behavioural Therapy (CBT), and mindfulness intervention. The major limitation is that none of the studies reported long-term results.

Blikman et al. [16] carried out a systematic review and meta-analysis published in 2013 of mild-high quality (AMSTAR 6). They performed research of literature until 2012 and selected 6 studies, all of which were RCTs or CCTs. They examined the efficacy of ECM (energy conservation treatment): education about balancing, modifying, and prioritizing activities, rest, self-care, effective communication, biomechanics, ergonomics, and environmental modifications. The authors declared that more high-quality RCTs are still needed.

Braley [17] published in 2010 a review with the aim of examining the most commonly proposed primary and secondary mechanisms of fatigue in MS, in particular for sleep specialists. The author reviewed tools for assessment of fatigue and available treatment approaches like energy conservation and relaxation therapy. The review has some methodologic limitations and is of low quality.

Yu et al. [14, 15] carried out a systematic review of mild quality (AMSTAR 6) that included 70 articles published until 2011. They classified the evidence in high, medium, and low quality and they selected 28 studies that reported intervention aimed at activity and participation. Eight studies concerned occupational therapy in fatigue management. They focused on three topics: face-to-face format like “Managing Fatigue” course and “Fatigue: Take Control” course, telerehabilitation, and Activity of Daily Living (ADL) training.

## 4. Discussion

Fatigue is thought to be a multidimensional symptom, it should be treated with a multidimensional approach targeting patients' behaviour as well as their emotional and mental attitude towards fatigue [27, 28]. All reviews supported a multidisciplinary treatment [7–13, 16].

NICE Guideline [7] recommend care for people with MS using a coordinated* multidisciplinary approach*, involving professionals who can best meet the needs of the person with MS and who have expertise in managing MS, including occupational therapist. Fatigue can be correlated with anxiety, depression, difficulty in sleeping, and any potential medical problems such as anaemia or thyroid disease. NICE guideline [7] recommends an assessment and treatment for this secondary fatigue. Fatigue management programme can be considered for treating MS-related fatigue, but no specific recommendation about occupational management of fatigue is reported in the guideline.

In a recent review Tur [9] reported that the multidisciplinary team, especially the occupational therapists and physiotherapists, once triggers of fatigue are identified, help the patient to set relevant and achievable goals, often related to instrumental activities of day-to-day life, before starting any therapeutic intervention.

The interventions of* energy conservation education programmes and fatigue management *try to help the patient to save energy through the implementation of different strategies such as work simplification or the use of labor-saving and ergonomic equipment [9, 11, 13, 16, 17].

These programs can be delivered in a group setting and they had good outcomes in terms of reducing the effects of moderate fatigue on the lives of people with MS [10]. These programs appear to improve quality of life (QoL) in people with MS in the short-term [10], but the quality of evidence is defined by NICE guideline [7] as low to very low. More high-quality RCTs are still needed to investigate the usefulness of these treatments in the longer-term [11].

A recent meta-analysis [13] that compared 8 RCTs focused on educational interventions, including self-management components (e.g., clients selecting strategies to manage fatigue based on their needs, environment, or preferences). They found significant improvement in six out of eight trials (75%) and the benefit was experienced also in a less homogeneous sample including older adults and those with progressive MS or with severe disability [13]. Another meta-analysis [16] showed that Energy Conservation Management (ECM) treatment was more effective than no treatment in improving subscale scores of the Fatigue Impact Scale, cognitive and psychosocial function, and QoL, especially in physical role, social function, and mental health. Limited or no evidence was found for the effectiveness of ECM treatment in the other outcomes in the short-term or mid-term. None of the studies reported long-term results. The best-evidence synthesis shows that there is limited evidence for the short-term effectiveness of ECM treatment over a support treatment reducing the impact of fatigue and in improving 3 QoL scales (role physical, social function, and mental health) in fatigued patients with MS.

Some reviews [9, 10] recommend also mixed programmes: FACETS was a very well designed randomized placebo-controlled trial with 164 MS patients. This study showed a beneficial effect of the FACETS programme, which consisted of six weekly sessions of around 90 minutes, on fatigue levels. This beneficial effect, with reduced fatigue severity and increased self-efficacy for managing fatigue, was observed after 1 and 4 months after finishing the intervention and it was still maintained at 1 year of follow-up [29–31].

Similarly, the EXIMS study was carried out in 120 patients with MS, who were randomly assigned to different groups: experimental group did 3-month exercise intervention plus usual care, and control group did usual care only. The primary objective was to evaluate the effect of a combined physical and psychological programme on the self-reported exercise behaviour. The authors found that the programme not only significantly improved the exercise behaviour but also was associated with a decrease in fatigue levels. The programme EXIMS involves a combination of supervised and nonsupervised home-base aerobic exercise and moderate resistance training. The supervised exercise sessions incorporate CBT, for example, setting goals, finding social support, and understanding the costs and benefits of exercise [9, 10].

The* behaviour change *interventions show benefit: participants in behaviour treatment are able to review their issues related to fatigue and develop skills to adjust their daily routine, activities, or environment to manage MS fatigue [12, 29, 30, 32, 33]. Common features of behaviour change interventions with significant effects are a minimum of six weeks of participation with one weekly session for no less than 50 min per session with trained interventionists as occupational therapists [12].

Also the* relaxation therapy* (RT) has been shown to have benefit, but the studies included have some limitations such as the use of a single therapist, potential underpowering, and exclusion of MS patients with EDSS scores > 6 [17].

The authors Yu and Mathiowetz [14, 15] reported that adapted equipment, training in self-care, and occupation-based therapeutic activities with functional training, such as a ADL training, can improve Functional Independence Measure and fatigue. Furthermore they reported a strong evidence supporting* face-to-face fatigue management programs* (“Managing Fatigue” course and “Fatigue: Take Control” course). These programs are courses, during 6 weeks, that encourage active engagement in occupations and emphasize balanced interaction between personal and environmental factors. The effects are the improvement of fatigue impact, self-efficacy, and quality of life and they were maintained in 1-year follow-up [14].

The author reported limited evidence about* online fatigue self-management course* (during 7 weeks) in teleconference. They reported a significant reduction in fatigue impact, but this improvement was no better than that attained by face-to-face programs [15].

The problem of fatigue and work instability is treated only by Khan [8] but the authors found insufficient evidence to support vocational rehabilitation programs in altering rates of job retention. However clinicians need to be aware of vocational issues and to understand and manage barriers to maintaining employment.

## 5. Conclusion

Considering the complex impact of fatigue in MS, it is necessary to provide a multidisciplinary rehabilitative approach that includes the role of OT. In particular, the efficacy of occupational interventions requires the adoption of fatigue self-management programs to teach patients ways of managing daily fatigue and energy conservation programs.

## Figures and Tables

**Table 1:** Inclusion and exclusion criteria used for paper selection.

Inclusion criteria	Exclusion criteria
Review or Systematic Review or Meta-analysis	Original study

Published last 10 years	Published before 10 years ago

English languages	Other languages

Full text available	Full text not available

Population with Multiple Sclerosis	Population with other diseases

Aged> 18 years old	Aged< 18 years old

Occupational rehabilitation	Other intervention

Fatigue management	Other symptoms management

**Table 2:** Tool used to assess quality of papers selected. Assessment of Multiple Systematic Reviews (AMSTAR)[7, 8].

(1) Was an ‘a priori' design provided?

(2) Was there duplicate study selection and data extraction?

(3) Was a comprehensive literature search performed?

(4) Was the status of publication (i.e. grey literature) used as an inclusion criterion?

(5) Was a list of studies (included and excluded) provided?

(6) Were the characteristics of the included studies provided?

(7) Was the scientific quality of the included studies assessed and documented?

(8) Was the scientific quality of the included studies used appropriately in formulating conclusions?

(9) Were the methods used to combine the findings of studies appropriate?

(10) Was the likelihood of publication bias assessed?

(11) Were the conflicts of interest stated?

**Table 3:** Included review characteristics and results. MS: multiple sclerosis; OT: occupational therapy; RCT: randomized clinical trial; SR: systematic review; CCT: controlled clinical trial, AMSTAR: Assessment of Multiple Systematic Reviews; QoL: quality of life: ADL: activity of daily living; EDSS: Expanded Disability Status Scale, FACETS: Fatigue: Applying Cognitive behavioural and Energy effectiveness Techniques to life Style.

Author	Type of study	Topic	Finding	Limits	AMSTAR
Khan [8]	Systematic review (15 Cochrane review and 24 other review)	(i) Multi disciplinary rehabilitation with OT (ii) FACETS programs (iii) Energy conservation (iv) Vocational therapy	Moderate evidence for TO	Lack of methodologically robust trials, 4 review included are of their group	10

Tur [9]	Review	(i) Energy conservation (ii) FACETS programs	Effective treatment to reduce fatigue	Not clear process of selection	2

Hourihan [10]	Review	(i) Energy conservation (ii) FACETS programs	Effective treatment to reduce fatigue	Not clear process of selection	2

Khan [11]	Systematic review (12 RCT 12 SR 2CCT 1 other; 6 about OT)	Energy conservation	Effective in reducing fatigue and improving QoL in short-term.	More high-quality RCTs are still needed	10

Asano [12]	Systematic review (38 RCT)	Fatigue management	Effective treatment to reduce fatigue	Small sample size	10

Asano et Finlayson [13]	Meta-analysis, (8 RCTs)	(i) Fatigue management program (ii) Energy conservation course (iii) Cognitive Behavioural Therapy (iv) Mindfulness intervention	Strong evidence for educational rehabilitation for reducing fatigue	None of the studies reported long-term results	6

Yu [14]; Yu [15]	Systematic review (70 trials)	(i) Face-to-face format (managing fatigue course and fatigue: take control course) (ii) Telerehabilitation (iii) ADL training	High effectiveness for face-to-face format; Low effectiveness for telerehabilitation	(i) All types of MS (ii) Limited evidence	4

Blikman [16]	Systematic review, meta-analysis 6 trials (4 RCTs and 2 CCTs)	(i) Energy conservation interventions	Effective reduction of fatigue in Short term and improved QoL	More high-quality RCTs are still needed	6

Bradley [17]	Review	(i) Energy conservation (ii) Relaxation therapy	Some benefit	(i) Use of a single therapist (ii) Potential underpowering, (iii) exclusion of patients with EDSS scores > 6	2

NICE [8]	Guideline	(i) Assessment (ii) Fatigue management programme (iii) Multidisciplinary team	Some benefit	Low quality of evidence	-
